# Supplementary material for: Molecular Genetic Characteristics of FANCI, a Proposed New Ovarian Cancer Predisposing Gene
Source: Genes (Basel). 2023 Jan 20;14(2):277. doi: 10.3390/genes14020277 (PMC9956348; doi:10.3390/genes14020277)
Supplement: Supplementary file 1 [file genes-14-00277-s001.zip › 09.11.22_Supplementary_figures_v2.docx]

PT0001


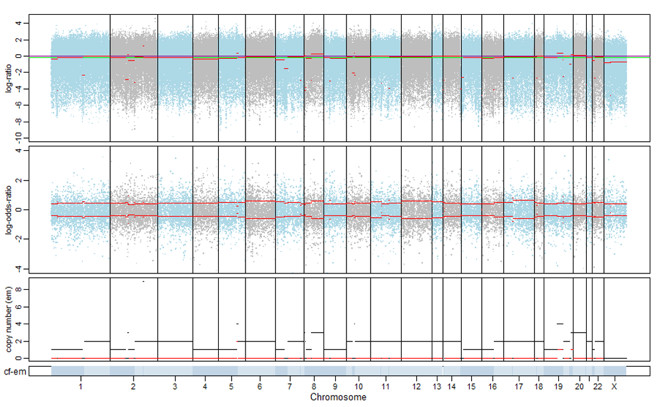


PT0002


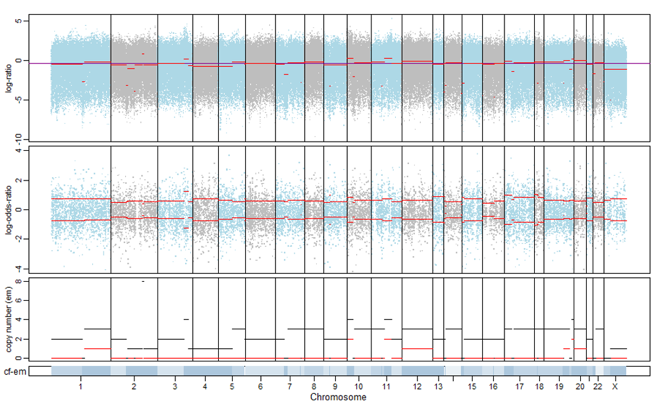


PT0003


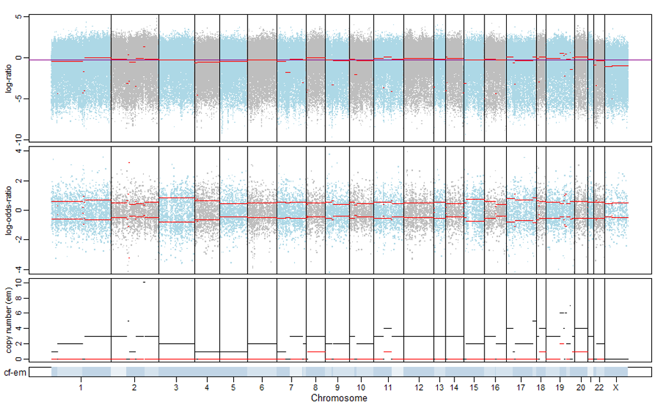


PT0004


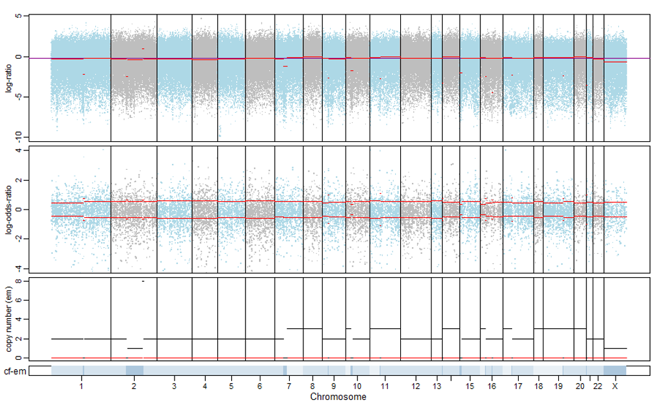


PT0006


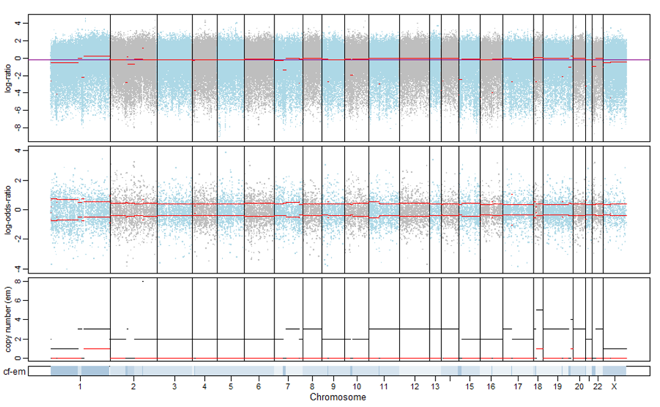


PT0005


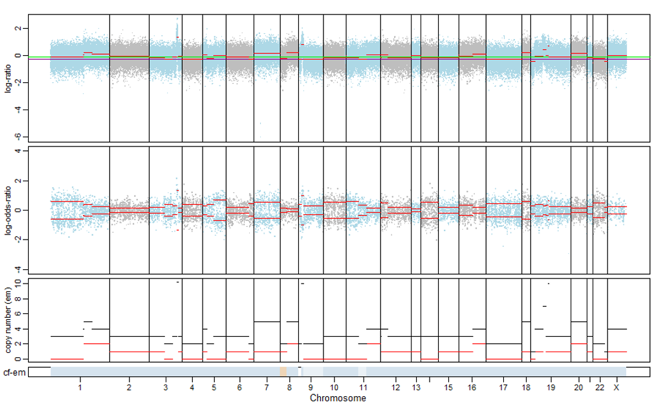


PT0007
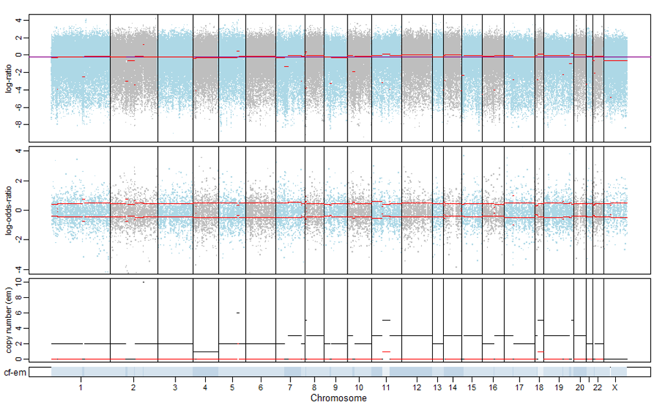


**Supplementary Figure 1.** Copy number alterations per chromosome across the genome of tumours from French Canadian cases harbouring germline *FANCI* c.1813C>T. The top panel shows total copy number log-ratio, the middle panel shows allele-specific copy number log-ratio, and the bottom panel shows the corresponding integer copy number calls (red is the minor allele).


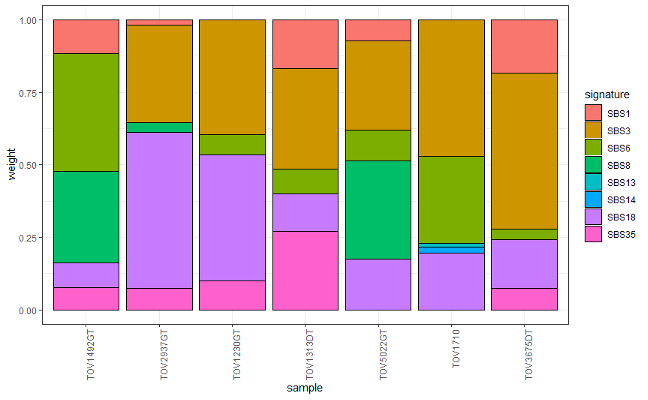


**Supplementary Figure 2.** Proportional representation of defined somatic mutational signatures observed in each ovarian tumour from French Canadian cases harbouring germline *FANCI* c.1813C>T.

SBS1: Ageing

SBS3: Homologous recombination deficiency

SBS6: Mismatch repair deficiency

SBS8: Unknown (putative homologous recombination deficiency)

SBS13: Activation of APOBEC family

SBS14: Polymerase epsilon mutation and mismatch repair deficiency

SBS18: Damage due to reactive oxygen species

SBS35: Platinum chemotherapy treatment

# Supplementary Figure 3. Pedigree of a Ukrainian breast and ovarian cancer family negative for *BRCA1* and *BRCA2* pathogenic variants where *FANCI* c.286G>A; p.E96K was identified by WES in the proband, indicated by the arrow. Age at diagnosis and death are shown where known. Cancer type (OC: ovarian, BC: breast, Pro: prostate, Col: colon, and Leu: leukemia) and age of diagnosis are shown.

DCIS: ductal carcinoma in situ

IDC: infiltrating ductal carcinoma
